# A Multitrait Analysis of Genome-Wide Association Study Reveals the Shared Genetic Architecture Between Inflammatory Bowel Disease and Ankylosing Spondylitis

**DOI:** 10.1155/mi/4012195

**Published:** 2025-10-30

**Authors:** Hongyan Li, Dadong Tang, Yanling Liu, Qijie Li, Hongchang Liu

**Affiliations:** ^1^Anorectal Disease Department, Hospital of Chengdu University of Traditional Chinese Medicine, Chengdu 610072, China; ^2^Clinical Medical College, Chengdu University of Traditional Chinese Medicine, Chengdu 611137, China; ^3^Anorectal Disease Department, Changshou Traditional Chinese Medicine Hospital, Chongqing 401220, China; ^4^Department of Surgical, Medical, Molecular Pathology and Critical Area, University of Pisa, Pisa, Italy

**Keywords:** AS, CPASSOC, genetic correlation, IBD, MTAG

## Abstract

**Background:**

Clinical evidence indicates that inflammatory bowel disease (IBD) and ankylosing spondylitis (AS) often co-occur, but their genetic mechanisms remain unclear. Our objective is to explore the genetic relationship between IBD and AS.

**Methods:**

Using large-scale summary statistics from genome-wide association study (GWAS), we investigated the shared genetic architecture between IBD, including ulcerative colitis (UC) and Crohn's disease (CD), and AS. Starting with genetic correlation, we then examined shared genetic structures and genes, followed by causal inference, and explored potential functional genes and biological pathways in tissue and cell types.

**Results:**

We observed a positive genetic correlation between IBD and AS (IBD–AS: *r*_*g*_ = 0.252, *p*=3.78e − 06; CD–AS: *r*_*g*_ = 0.268, *p*=5.19e − 06; UC–AS: *r*_*g*_ = 0.171, *p*=6.64e − 03). Multitrait analysis of GWAS (MTAG) and cross-phenotype association analysis (CPASSOC) identified 24 pleiotropic single-nucleotide polymorphisms (SNPs) across three trait pairs. Gene association analysis from three methods collectively identified eight shared functional genes for IBD and AS. Shared tissue-specific genetic enrichment was found in lung, spleen, small intestine, and whole blood tissues. Additionally, common enrichment was observed in specific cell types, such as T and B cells. Bidirectional Mendelian randomization (MR) analysis revealed no causal relationship between the two conditions.

**Conclusions:**

This study confirms the genetic correlation between IBD and AS, identifies their shared genetic architecture and biological pathways, providing strong evidence for the genetic comorbidity of IBD and AS. These findings offer directions for future research.

## 1. Introduction

Inflammatory bowel disease (IBD) and ankylosing spondylitis (AS) are both characterized by recurrent inflammation, with clinical symptoms often overlapping in the gut and joints [1, 2]. Arthritis is a typical extra-intestinal manifestation of IBD, with one-third of IBD patients exhibiting AS-related joint features [3, 4]. Similarly, intestinal inflammation is a common extra-articular symptom of AS, with ~10% of AS patients presenting with IBD-related gastrointestinal symptoms [5]. A large body of previous research has confirmed the comorbid relationship between IBD and AS from clinical features, epidemiological data, and pathogenesis perspectives [6–8].

IBD and AS exhibit a high degree of genetic heritability. A random sample from Iceland revealed that the risk of developing either or both diseases was significantly increased among third-degree relatives. The sibling recurrence risk of IBD was found to be 3.0 times higher in first-degree relatives of AS probands [9]. It has been reported that IBD and AS show a significant genetic link in the major histocompatibility complex (MHC) region [10, 11], and previous studies have identified several independent genetic variants in both MHC and non-MHC regions that are associated with susceptibility to IBD and AS [12–15]. However, the complex pathogenesis between IBD and AS remains incompletely understood, and investigating the mechanisms underlying the co-occurrence of intestinal and joint manifestations has become the focus of our current research.

**Figure 1:**
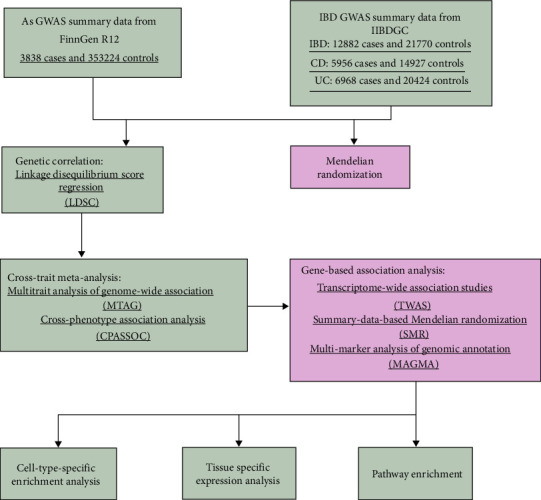
Flowchart of this study. AS, ankylosing spondylitis; CD, Crohn's disease; IBD, inflammatory bowel disease; UC, ulcerative colitis.

Based on previous genome-wide association study (GWAS) studies, we employed several genetic methods to enrich the comorbid genetic features between IBD and AS. Linkage disequilibrium score regression (LDSC) was used to estimate the genetic correlation between IBD and AS; multitrait analysis of GWAS (MTAG) and cross-phenotype association analysis (CPASSOC) were applied to identify pleiotropic loci; three gene-based association methods were used to determine potential shared genes, which were subsequently enriched in specific tissues and cell types; and Mendelian randomization (MR) was used for causal inference. Our goal was to identify shared single-nucleotide polymorphisms (SNPs) and genes between IBD and AS, as well as enriched pathways, tissues, and cell types, to explore the shared genetic architecture between IBD and AS. The overall workflow of this study is shown in Figure 1.

## 2. Materials and Methods

### 2.1. Data

Regarding the characteristics of IBD, we included GWAS data for IBD, ulcerative colitis (UC), and Crohn's disease (CD) traits. The GWAS data for the three traits were sourced from the International Inflammatory Bowel Disease Genetics Consortium (IIBDGC). Specifically, the IBD dataset included 21,770 controls and 12,882 cases, the CD dataset consisted of 14,927 controls and 5956 cases, and the UC dataset comprised 20,464 controls and 6968 cases. The GWAS data for AS were obtained from the latest r12 version of the FinnGen study, which includes 3838 cases and 353,224 controls. All populations in these datasets were of European ancestry. Additionally, cis-eQTL summary data were obtained from the genotype-tissue expression (GTEx) project v8 [16] for four tissues (Colon_Sigmoid, Colon_Transverse, Small_Intestine_Terminal_Ileum, and Whole_Blood), containing SNPs located within 1 Mb of the transcription start site.

### 2.2. LDSC

LDSC analysis quantifies the genetic correlation between two traits by utilizing GWAS summary statistics. This method estimates the genetic contributions of complex diseases and traits while avoiding bias introduced by sample overlap. By examining the relationship between the linkage disequilibrium (LD) score and the SNP statistics in GWAS results, LDSC can quantify the independent contributions of polygenic effects. Additionally, chi-square statistics are used to detect deviations from the null hypothesis, revealing genetic correlations [17]. We used precomputed LD scores and weights based on the European population from the 1000 Genomes Project to calculate both the heritability of individual traits and the genetic correlations between traits [18]. Heritability values for individual traits range from 0 to 1, with values closer to 1 indicating stronger genetic effects. Genetic correlation (rg) between traits is positive when they are positively correlated and negative when they are negatively correlated.

### 2.3. Cross-Trait Meta-Analysis

We identified pleiotropic loci between IBD, UC, CD, and AS through MTAG and CPASSOC. MTAG [19] extends the inverse variance-weighted meta-analysis by utilizing summary statistics from single-trait GWAS to refine the effect size estimates for each trait. It achieves this by incorporating relevant information from other traits, thereby boosting the statistical power to detect genetic associations. Additionally, bivariate LDSC was employed to address potential sample overlap between the GWAS results of different traits and to generate trait-specific effect estimates for each SNP. CPASSOC [20] integrates summary statistics from multiple traits to detect shared variants, providing two test statistics, SHom and SHet. SHom is based on a fixed-effect meta-analysis approach and considers data correlation between traits and cohorts, which may arise from relatedness, potential overlap, or sample correlation. SHet is an extension of SHom, allowing for heterogeneity in trait effects across different designs, environments, or populations, as well as across different phenotypes. Thus, we used SHet as the test statistic for CPASSOC analysis. Significant pleiotropic SNPs between traits were defined as variants with *P*_MTAG_ and *P*_CPASSOC_ < 5e−8. Subsequently, independent SNPs were obtained through LD clumping analysis using PLINK, with parameters set to *R*^2^ < 0.001 within a 10,000 kb window. Finally, we performed gene functional annotation of pleiotropic SNPs identified by MTAG and CPASSOC using ANNOVAR [21].

### 2.4. Gene-Based Association Analysis

We applied transcriptome-wide association studies (TWAS), summary-data-based MR (SMR), and multimarker analysis of genomic annotation (MAGMA) to identify shared genes between IBD, UC, CD, and AS. The input data for the three gene-level analyses were derived from the GWAS summary statistics after MTAG analysis. The significance level of false discovery rate (FDR) < 0.05, after correction for FDR using Bonferroni correction, was considered statistically significant.

TWAS analysis was performed using functional summary-based imputation (FUSION) [22], which develops predictive models for the genetic components of functional/molecular phenotypes and associates input gene expression with traits using GWAS summary data to identify significant trait-gene associations. We conducted TWAS using expression models from the GTEx project v8, including four tissues (Colon_Sigmoid, Colon_Transverse, Small_Intestine_Terminal_Ileum, and Whole_Blood).

SMR analysis was utilized to explore causal relationships between eQTLs and GWAS outcomes. We performed SMR using cis-eQTL summary data from four relevant tissues (Colon_Sigmoid, Colon_Transverse, Small_Intestine_Terminal_Ileum, and Whole_Blood) from GTEx v8. In the SMR analysis, a 1000 kb window upstream and downstream of the top eQTL was considered the cis region, and related eQTLs that met the threshold (*p* < 5e − 8) were selected as instrumental variables. SNPs with an allele frequency exceeding the specified threshold (set to 0.2) were excluded. HEIDI [23] was then applied to test for pleiotropy and linkage. A *p*-value < 0.01 in the HEIDI test indicated the potential existence of pleiotropy [24].

The MAGMA [25] method is designed for gene- or gene-set-based association analysis, directly identifying functional genes or modules associated with the trait of interest. MAGMA's gene analysis utilizes a multivariate linear principal component regression model, where the SNP matrix of genes is initially projected onto its principal components (PCs), which are then used as predictors in a linear regression model for phenotypes. This approach enables MAGMA to analyze continuous genetic traits and expand to joint and interaction analyses of multiple gene sets and other genetic traits. For our analysis, we used the European ancestry panel from the 1000 Genomes Project as the LD reference.

Finally, we merged the results from the three gene-level association analyses to derive a set of shared genes for each trait pair.

### 2.5. Pathway Enrichment

We performed pathway enrichment analysis using GO and KEGG to investigate the functions, mechanisms, and interactions of the shared genes identified through gene-based association analysis, in order to understand their roles in biological processes.

### 2.6. Tissue and Cell-Specific Analysis

Tissue-specific expression analysis (TSEA) is used to investigate gene expression patterns across different tissues and to reveal the potential functions of genes in biological processes [26]. We conducted tissue-specific expression analysis of shared genes using the “deTS” package based on RNA-seq data from the GTEx database. Additionally, we performed cell-type-specific enrichment analysis (CSEA) of these genes using WebCSEA [27]. WebCSEA is a fast network application developed by Dai et al. for assessing gene specificity in different cell types (https://bioinfo.uth.edu/WebCSEA/). The tool integrates single-cell RNA sequencing data from 111 human tissues to 1355 tissue cell types from 11 human organ systems and has optimized the deTS algorithm to evaluate the enrichment of each cell type. Finally, we applied the Bonferroni correction to control the FDR (FDR < 0.05).

### 2.7. MR

We assessed the causal association between IBD and AS through a bidirectional two-sample MR analysis. Four MR analysis methods, including IVW, weighted median, maximum likelihood, and MR–Egger, were employed, with IVW as the primary method and the other methods used for sensitivity analysis. We used SNPs as instrumental variables and set a threshold of *p* < 5e − 8 for strong association SNPs. The European population from the 1000 Genomes Project was used as the reference, and LD checks were performed under the conditions of *R*^2^ = 0.001 and a window size of 10,000 kb. Additionally, we utilized PhenoScanner (http://phenoscanner.medschl.cam.ac.uk/) to exclude SNPs associated with the outcome or its risk factors. Cochran's *Q* test was applied to assess heterogeneity, and MR–Egger intercepts were used to examine horizontal pleiotropy.

## 3. Results

### 3.1. Genetic Correlations

We calculated the SNP-based genetic correlation of IBD and AS through LDSC. The results are shown in Table 1. The genetic estimates for individual traits indicate that both IBD, CD, and AS have genetic effects. The genetic correlation estimates between traits revealed a positive genetic correlation among IBD, UC, CD, and AS, with significant associations (*p* < 0.05). Notably, the low and nonsignificant SNP-based heritability of UC may explain its weaker genetic correlation with AS compared to CD.

### 3.2. Identification of Pleiotropic Loci

**Figure 2:**
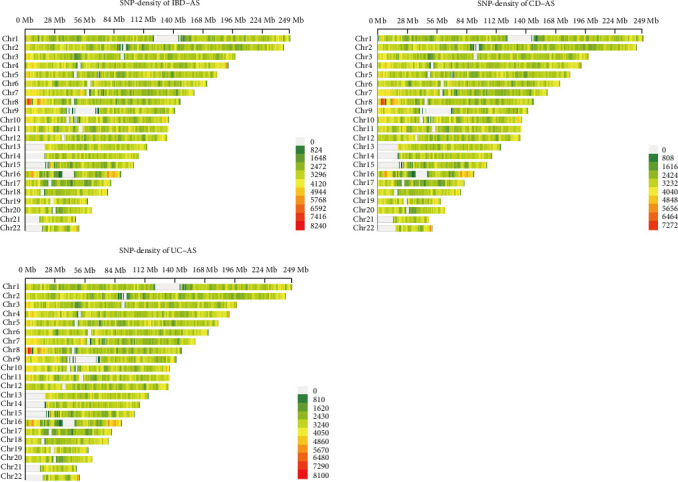
Density plot of SNP distribution for AS and IBD.

Given the significant genetic correlations between AS and IBD, we next sought to identify the specific pleiotropic loci driving this association and to investigate any potential causal relationships. MTAG identified 6269 significant SNPs for IBD (*p* < 5e − 8), 5053 significant SNPs for CD, and 2754 significant SNPs for UC (Tables S1–S3).  Figure 2 illustrates the specific distribution density of these SNPs. CPASSOC identified 15,371 significant SNPs for the IBD–AS trait pair, 14,146 significant SNPs for the CD–AS trait pair, and 11,055 significant SNPs for the UC–AS trait pair (Tables S4–S6). By combining MTAG and CPASSOC, we identified 24 significant pleiotropic SNPs shared across the three traits, with 14 located in the MHC region and 10 in non-MHC regions (Table 2). The non-MHC pleiotropic loci include five SNPs (rs11209026, rs10800756, rs4676408, rs348595, and rs13048321) significantly associated with IBD–AS traits; three SNPs (rs7547569, rs12132298, and rs2836883) significantly associated with UC–AS cross-trait GWAS; and three SNPs (rs11209026, rs12131796, and rs17234657) significantly associated with CD–AS traits. Notably, rs11209026 was significantly associated with both IBD–AS and CD–AS traits (Table 2). Gene functional annotation of the pleiotropic SNPs using ANNOVAR revealed that PSMG1 is the nearest gene for two pleiotropic SNPs in IBD–AS and UC–AS traits. Similarly, PTGER4 is the nearest gene for two pleiotropic SNPs in IBD–AS and CD–AS traits. Additionally, IL23R is closest to two SNPs shared by all three trait pairs. The remaining pleiotropic SNPs are associated with INAVA, GPR35, and CACNA1S (Table 2).

### 3.3. Shared Genes and Pathways

**Figure 3:**
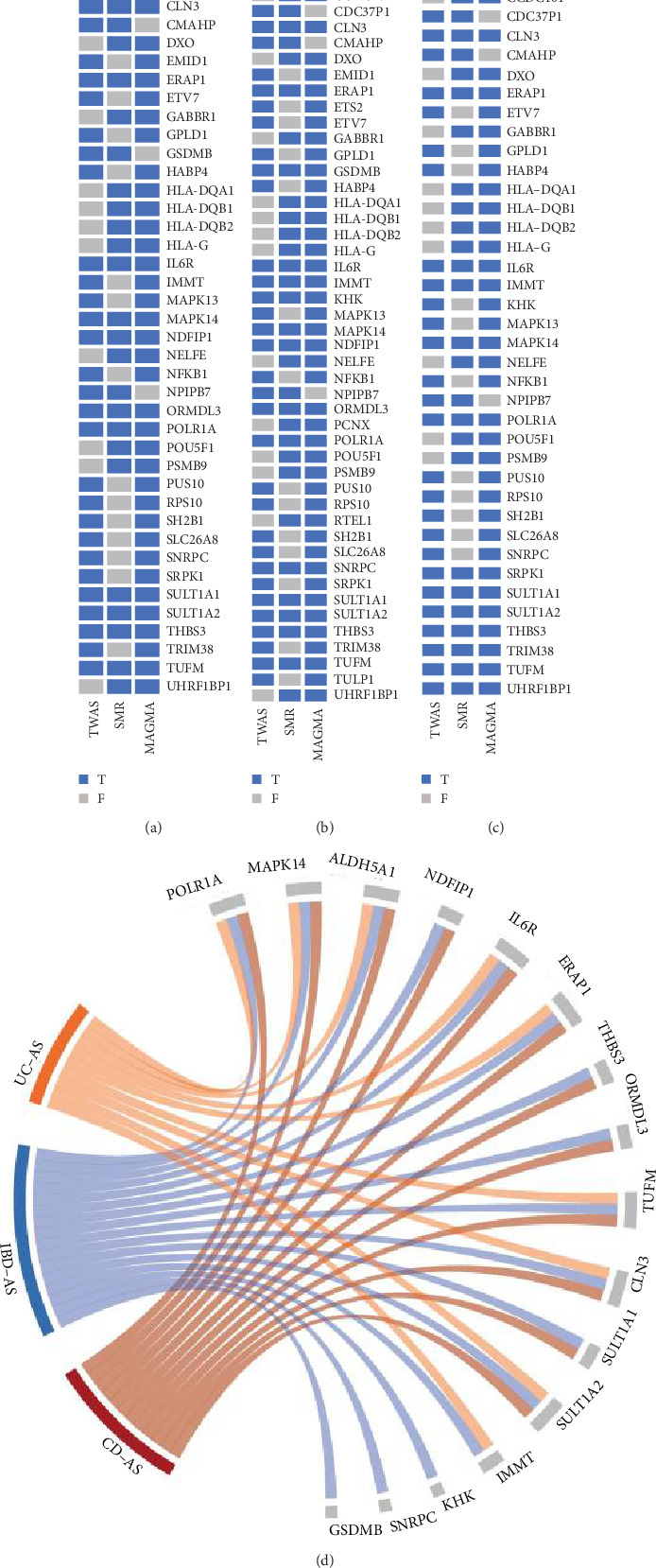
Three association analyses identify shared genes for trait pairs: (A) shared genes for CD–AS trait pairs; (B) shared genes for IBD–AS trait pairs; (C) shared genes for UC–AS trait pairs; and (D) significantly shared genes identified by three methods. *T*, significant; *F*, nonsignificant; AS, ankylosing spondylitis; CD, Crohn's disease; IBD, inflammatory bowel disease; MAGMA, multimarker analysis of genomic annotation; SMR, summary-data-based Mendelian randomization; TWAS, transcriptome-wide association studies; UC, ulcerative colitis.

**Figure 4:**
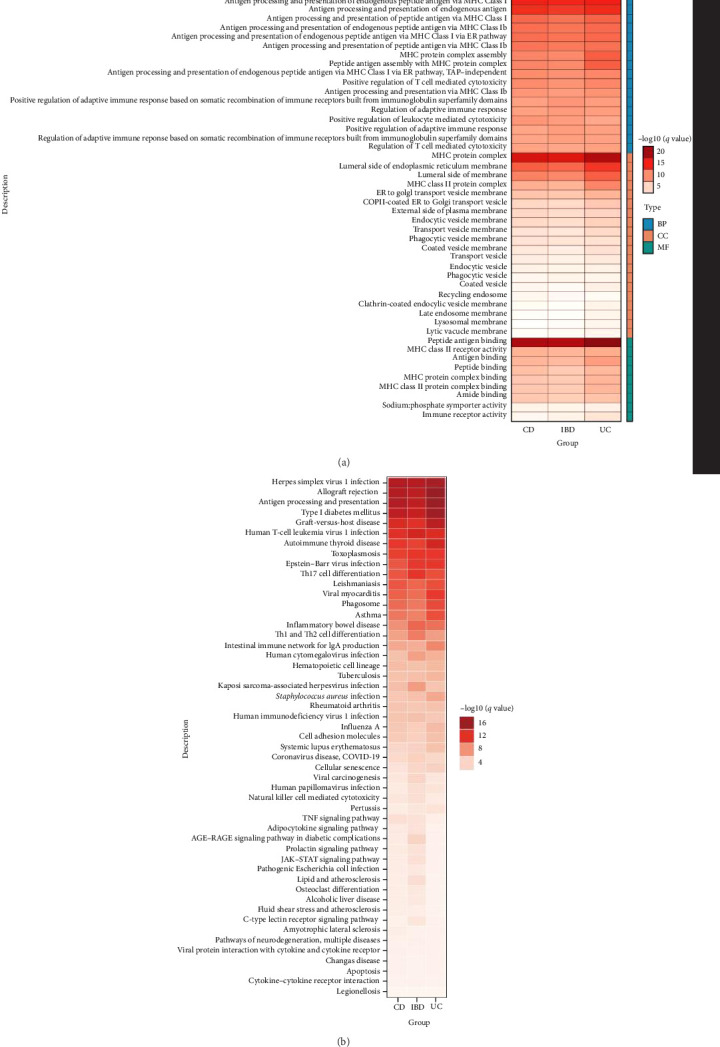
Enrichment analysis results: (A) GO enrichment analysis results and (B) KEGG enrichment analysis results.

The annotation of shared SNPs and their nearby genes appeared overly simplistic, failing to fully explain the pleiotropy. Therefore, we applied three methods, TWAS, SMR, and MAGMA, to identify shared genes between IBD (including UC and CD) and AS. In the three trait pairs, we identified a total of 1220 genes (at least one method was significant) (Table S7). Further, in the CD–AS trait pair, 45 genes were identified as significant through at least two methods (Figure 3A), while 52 genes were associated with the IBD–AS trait pair through at least two methods (Figure 3B), and 43 genes were identified in the UC–AS trait pair (Figure 3C). Lastly, integrating the results, 12 genes were shared among all three methods for the CD–AS trait pair (Figure 3A), 16 for the IBD–AS trait pair (Figure 3B), and 8 for the UC–AS trait pair (Figure 3C). Among these, MAPK14, POLR1A, IL6R, SULT1A2, TUFM, ERAP1, ALDH5A1, and CLN3 were shared across all three methods and all three trait pairs (Figure 3D). Gene enrichment analysis of the shared genes revealed that their functions are mainly involved in antigen processing and presentation, peptide antigen binding, MHC protein complexes, and various immune-inflammatory diseases (Figure 4).

### 3.4. Tissue-Specific Expression and Cell Type Enrichment

**Figure 5:**
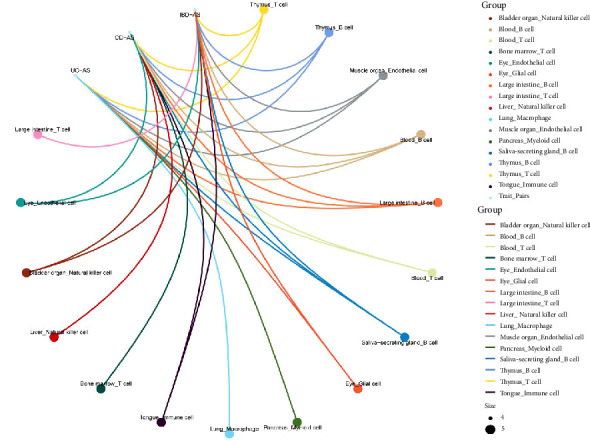
Cell-type-specific enrichment analysis. AS, ankylosing spondylitis; CD, Crohn's disease; IBD, inflammatory bowel disease; UC, ulcerative colitis.

Based on GTEx tissue enrichment analysis of the shared genes, we found significant enrichment in small intestine, spleen, lung, and blood tissues for all three trait pairs (Table 3). Furthermore, thymus T cells, thymus B cells, muscle endothelial cells, blood B cells, and colon B cells were significantly enriched across all three trait pairs (Figure 5). Additionally, pancreatic bone marrow cells and lung macrophages were exclusively enriched in the UC–AS pair, while bone marrow T cells and liver natural killer cells were exclusively enriched in the CD–AS pair. Collectively, these enriched tissues and cell types, together with the functional enrichment, highlight the pivotal role of immune-inflammatory processes in the pathogenesis of IBD and AS.

### 3.5. MR Analysis

**Figure 6:**
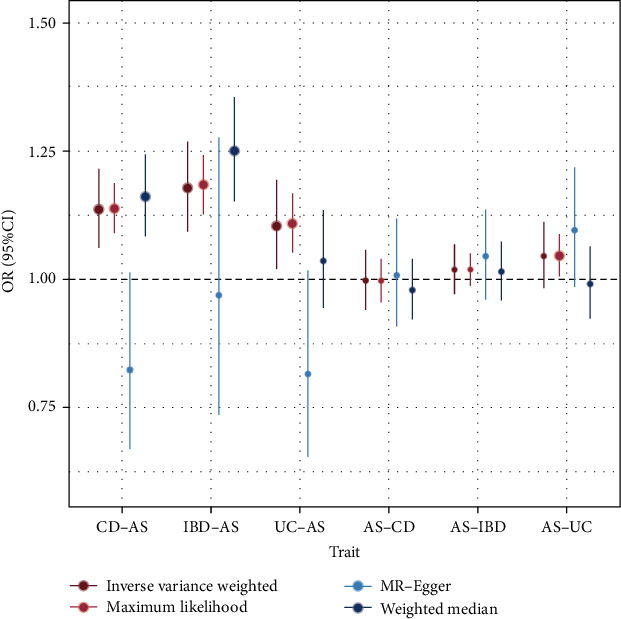
Results of bidirectional Mendelian randomization analysis. AS, ankylosing spondylitis; CD, Crohn's disease; IBD, inflammatory bowel disease; UC, ulcerative colitis.

We performed bidirectional MR to infer causal relationships between IBD (including UC and CD) and AS. No causal effect of IBD, CD, or UC on AS was observed. Although the IVW method showed strong significance (*p*_IVW_  < 0.05), the MR–Egger results were not significant, and the direction of effects was opposite to that of IVW. Therefore, we do not consider IBD, UC, or CD to have a causal effect on AS (Figure 6 and Table S8). In the reverse MR analysis, no causal effect of AS on IBD, UC, or CD was observed, with specific results presented in Figure 6 and Table S8.

## 4. Discussion

In this study, we identified shared genetic structures between IBD and AS through GWAS summary statistics. Further analyses explored their shared genes, biological pathways, and the traits' associations with shared cell and tissue types, emphasizing pleiotropy rather than causal relationships. Our findings contribute to advancing the understanding of the common genetic etiology between IBD and AS.

Many epidemiological studies have reported comorbid associations between these two diseases [7, 28], but a comprehensive genetic association analysis between IBD and AS has not yet been conducted. This study, based on large-scale datasets and the latest GWAS summary data, provides reliable genetic explanations for the shared genetic factors between IBD and AS. Our results demonstrate a strong genetic correlation between IBD and AS. Given their genetic correlation, we performed MTAG and CPASSOC to identify shared SNPs that were significantly associated with both IBD and AS. Previous studies have shown that both IBD and AS exhibit strong genetic effects in the MHC region [29, 30]. In this study, 14 shared SNPs were found in the MHC region, and functional enrichment analyses indicated involvement of the MHC protein complex pathway. Additionally, 10 SNPs associated with non-MHC regions were identified, which play a role in regulating shared pathways between IBD and AS. Among these, rs11209026 located at the IL23R locus exhibited the strongest significance (*p*=3.31e − 69), and it has been widely reported in the genetic associations of both IBD and AS [31, 32]. The C allele of rs11209026 is significantly associated with an increased risk of IBD, leading to overactivation of IL23R, which promotes cytokine stimulation of intestinal epithelial cells, fibroblasts, and endothelial cells, resulting in the secretion of more proinflammatory cytokines and chemokines, thereby recruiting immune cells (such as macrophages and T cells) to enhance the inflammatory response [33, 34]. In AS, proinflammatory cytokines activated by IL-23R stimulate fibroblasts and chondrocytes to secrete matrix metalloproteinases, accelerating the destruction of bone and joint tissues [35, 36]. Furthermore, among the 10 non-MHC loci, rs10800756 located at the CACNA1S locus has not previously been reported to be genetically associated with AS and IBD, making it a novel locus identified in this study. The protein encoded by the CACNA1S gene is a subunit of the calcium ion channel, involved in regulating intracellular calcium ion concentrations [37]. rs10800756 may influence the function of calcium channels, altering cellular responses to calcium signals. In IBD, this variation may enhance calcium ion influx into T cells or macrophages, increasing the secretion of inflammatory factors [38]. Additionally, calcium signaling is involved in the functional regulation of epithelial cells by affecting tight junction proteins in intestinal epithelial cells, disrupting the intestinal barrier, and triggering immune responses and inflammation [39]. However, in AS patients, bone fusion and bone destruction are among the ultimate pathological outcomes. Inflammatory cytokines secreted by immune cells and pathways regulating calcium signaling work together to accelerate bone resorption and disrupt bone remodeling [40]. CACNA1S may interfere with osteocytes' response to calcium signals, thereby exacerbating bone calcification and sclerosis.

Given the strong genetic correlation between IBD and AS and the presence of pleiotropic genetic variants, we investigated the causal relationship between them. Our findings suggest that the relationship between IBD and AS is likely driven by genetic pleiotropy rather than a causal relationship. Although previous MR analyses have reported causal associations between them [41, 42], potential biases may have arisen due to pleiotropy and confounding factors that were not fully accounted for. Additionally, inconsistencies could stem from the diversity and one-sidedness of the data used.

Through a series of genetic association analyses, we identified eight significantly shared genes (MAPK14, POLR1A, IL6R, SULT1A2, TUFM, ERAP1, ALDH5A1, and CLN3) that are associated with both IBD and AS. These genes are potentially involved in the pathogenesis of both diseases. The ERAP1 (Endoplasmic Reticulum Aminopeptidase 1) gene encodes an enzyme located in the endoplasmic reticulum, which is a member of the aminopeptidase family [43]. Polymorphisms in the ERAP1 gene may serve as risk factors for the development of AS and IBD [44], and its association with AS has been confirmed across multiple population cohorts [45, 46]. Animal studies [47] have shown that ERAP1-/- mice exhibit a decrease in type 1 regulatory T cells and display characteristic skeletal features of AS, including spinal ankylosis and inflammation. These mice also demonstrate increased susceptibility to spontaneous gut dysbiosis and colitis. Furthermore, research suggests that inhibition of ERAP1 activity may have therapeutic potential in AS patients, as ERAP1 may influence inflammatory and immune pathways by participating in processes such as angiogenesis and macrophage activation [48, 49]. MAPK14, also known as p38α, encodes p38 mitogen-activated protein kinase (MAPK), a member of the MAPK family widely expressed in eukaryotic cells [50]. In macrophages, MAPK14 is involved in the synthesis of proinflammatory cytokines [51], such as TNF-α, IL-1β, and IL-6, which play a crucial role in local inflammatory responses. The functional importance of p38α in inflammatory diseases has been extensively reported [52], with it being considered a key mediator of IBD-related inflammation, exhibiting the highest activity in the intestinal mucosa of IBD patients [50]. Additionally, previous studies have confirmed that MAPK14 is a central therapeutic target for both IBD and AS [53, 54]. The IL6R gene encodes the interleukin 6 receptor (IL-6R), a key protein involved in the binding of the IL-6 cytokine to its receptor, thereby mediating biological effects [55]. IL-6R participates in immune responses, inflammatory reactions, and various physiological and pathological processes. It is known to be associated with inflammatory diseases, such as UC and CD [56]. Polymorphisms in the IL6R gene have been reported to be associated with IBD in both cohorts [57, 58]. Additionally, IL6R is implicated in the pathogenesis of AS, with studies suggesting that IL6R could serve as a diagnostic biomarker for AS [59], and the inhibition of IL-6R reduces the risk of AS [60]. The TUFM gene encodes the mitochondrial translation elongation factor, an essential translation factor in mitochondria that plays a crucial role in protein synthesis. It binds to mitochondrial transfer RNA (tRNA) and facilitates the transfer of amino acids, thereby aiding in peptide chain elongation [61]. TUFM mediates the activation of the LAP-induced MAPKs-AP1 signaling pathway, triggering an inflammatory cytokine response [62]. Additionally, the role of TUFM in maintaining mitochondrial function and regulating immune cell activity is disrupted, leading to abnormal activation of the immune system, which induces chronic inflammation and autoimmune responses [63]. This may be associated with the immune inflammation seen in IBD and AS.

At the gene-tissue-cell level, our gene function analysis further revealed a shared biological hypothesis between IBD and AS, with their common biological mechanisms primarily involving immune pathways and inflammatory responses. The enrichment of shared genes in lung and spleen tissues suggests that their shared pathways may extend to other organs and tissues. This finding is consistent with previous studies that reported significant enrichment of IBD, CD, and UC in various immune-related tissues [64]. IBD may lead to a range of pulmonary diseases [65, 66], such as interstitial lung disease, granulomatous lung disease, and obstructive lung disease. MR analysis provides causal links between IBD and interstitial lung disease [67], as well as imaging evidence of altered pulmonary characteristics in IBD patients [68]. These associations may be established through pathways involving microbiota and their metabolites in the gut-lung axis, as well as innate and adaptive immunity [69]. Similarly, there is a close relationship between AS and lung tissue characteristics [70, 71], with early diffuse parenchymal changes observed in AS patients [72]. On the other hand, the spleen, as an essential component of the immune system, may potentially influence the onset and progression of both IBD and AS. Previous studies have reported the correlation between spleen size in IBD and the efficacy of spleen-targeted drug therapies based on its immune function for treating IBD [73, 74]. The shared enrichment of IBD and AS in lung and spleen tissues underscores the critical role of immune tissues and immune dysfunction in their pathogenesis.

Our genetic study provides evidence for the shared biological mechanisms between IBD and AS, offering direction for the prevention and treatment of IBD–AS comorbidities. This study has several limitations. Firstly, the datasets were restricted to individuals of European ancestry, which may limit the applicability of the findings to other populations. Secondly, while potential shared genetic mechanisms were identified, these results may be influenced by the limitations of the existing databases, and further research is needed to explore the shared biological pathways. Third, our MR analysis may still be subject to limitations related to sample size and genetic variation, which could lead to differences in the overall results.

## 5. Conclusions

In conclusion, this study confirms the genetic association between IBD and AS and identifies their shared genetic structure as well as biological pathways, providing strong evidence for and the basis of comorbidity between IBD and AS. These findings provide directions for more in-depth studies in the future.

## Figures and Tables

**Table 1:** Heritability and genetic correlation results for AS and IBD (including CD and UC) estimated using LDSC.

Single trait	Heritability (*h*^2^)	Heritability (*h*^2^, *p*)	Trait1–Trait2	Genetic correlation (*r*_*g*_)	Genetic correlation (*r*_*g*_, *p*)
AS	0.388	4.98e−15	IBD–AS	0.252	3.78e−06
IBD	0.277	3.89e−19	CD–AS	0.268	5.19e−06
CD	0.224	1.09e−11	UC–AS	0.171	6.64e−03
UC	0.022	1.25e−01	—	—	—

Abbreviations: AS, ankylosing spondylitis; CD, Crohn's disease; IBD, inflammatory bowel disease; LDSC, linkage disequilibrium score regression; UC, ulcerative colitis.

**Table 2:** Pleiotropic loci for IBD–AS trait pairs from MTAG and CPASSOC analyses.

SNP	Trait1	Trait2	Chr	BP	A1	A2	MTAG trait1	MTAG trait2	CPASSOC	Nearest gene
rs11209026	IBD	AS	1	67705958	A	G	3.31e−69	3.99e−17	3.41e−70	IL23R
rs10800756	IBD	AS	1	201024059	A	G	5.37e−12	4.80e−10	2.85e−09	CACNA1S
rs4676408	IBD	AS	2	241574401	A	G	4.29e−13	1.75e−08	7.31e−11	GPR35
rs348595	IBD	AS	5	40323714	G	A	1.58e−27	2.40e−10	5.85e−26	PTGER4
rs72834678	IBD	AS	6	26108375	A	G	3.87e−12	6.23e−117	4.14e−131	—
rs6914981	IBD	AS	6	27556113	A	G	1.65e−09	3.69e−89	1.07e−99	—
rs2233965	IBD	AS	6	31080899	G	T	2.01e−22	7.16e−130	2.09e−143	—
rs2442725	IBD	AS	6	31319896	C	T	1.58e−13	4.01e−248	4.59e−287	—
rs2516427	IBD	AS	6	31471454	C	T	3.30e−08	1.43e−74	3.51e−83	—
rs114027542	IBD	AS	6	32602231	T	C	4.87e−11	9.59e−123	2.38e−138	—
rs13048321	IBD	AS	21	40459092	T	C	2.66e−22	8.73e−09	8.52e−21	PSMG1
rs11209026	CD	AS	1	67705958	A	G	3.12e−59	7.58e−16	6.72e−58	IL23R
rs12131796	CD	AS	1	200878727	A	G	9.88e−09	1.03e−10	6.70e−10	INAVA
rs17234657	CD	AS	5	40401509	G	T	3.95e−31	3.63e−09	6.85e−30	PTGER4
rs186640245	CD	AS	6	31224308	G	A	5.82e−11	7.91e−178	7.25e−204	HLA-C
rs2534684	CD	AS	6	31462672	C	A	2.89e−09	1.54e−75	7.01e−84	MICB
rs75084410	CD	AS	6	32636018	A	G	1.90e−08	1.08e−122	7.22e−140	HLA-DQB1
rs2071536	CD	AS	6	32821447	T	C	3.01e−09	3.38e−68	2.49e−75	TAP1
rs7547569	UC	AS	1	67731368	C	T	3.85e−27	3.12e−09	1.69e−25	IL23R
rs12132298	UC	AS	1	200875095	C	T	1.55e−14	1.42e−10	9.69e−12	INAVA
rs2227228	UC	AS	6	28463576	C	T	9.50e−10	1.56e−81	3.14e−90	GPX6
rs1055890	UC	AS	6	31321915	G	A	2.10e−08	7.97e−249	5.33e−284	—
rs4947328	UC	AS	6	31561747	G	A	3.73e−11	3.08e−53	3.34e−58	—
rs9271385	UC	AS	6	32587409	G	A	1.83e−18	1.47e−11	7.05e−29	—
rs2836883	UC	AS	21	40466744	A	G	1.96e−23	4.08e−09	1.59e−21	PSMG1

*Note:* A1, effect allele; A2, other allele.

Abbreviations: AS, ankylosing spondylitis; BP, base pair; CD, Crohn's disease; Chr, chromosome; CPASSOC, cross-phenotype association; IBD, inflammatory bowel disease; MTAG, multitrait analysis of genome-wide association study; SNPs, single-nucleotide polymorphisms; UC, ulcerative colitis.

**Table 3:** Significant results of TSEA.

Trait pair	Tissues	*p*_FDR
IBD–AS	Lung	3.73e−02
	Small intestine: terminal ileum	2.21e−03
	Spleen	3.15e−06
	Uterus	1.49e−02
	Whole blood	1.86e−02

UC–AS	Lung	4.67e−02
	Small intestine: terminal ileum	5.42e−03
	Spleen	3.02e−06
	Uterus	1.49e−02
	Whole blood	2.01e−02

CD–AS	Lung	4.67e−02
	Small intestine: terminal ileum	4.42e−03
	Spleen	2.85e−05
	Uterus	1.52e−02
	Whole blood	1.86e−02

Abbreviations: AS, ankylosing spondylitis; CD, Crohn's disease; IBD, inflammatory bowel disease; TSEA, tissue-specific expression analysis; UC, ulcerative colitis.

## Data Availability

The GWAS data for IBD, UC, and CD were obtained from the International Inflammatory Bowel Disease Genetics Consortium (https://www.ibdgc.org/). The GWAS data for AS were sourced from the R12 version of the FinnGen database (https://r12.finngen.fi/). The GTEx v8 multitissue expression weight data used in the TWAS were retrieved from the FUSION website (http://gusevlab.org/projects/fusion/). The eQTL summary data for multiple tissues were downloaded from GTEx (https://gtexportal.org/home/downloads/adult-gtex/qtl). Reference genes in MAGMA were obtained from FUMA GWAS (https://fuma.ctglab.nl/downloadPage).

## Supporting Information

Supporting Information Table S1: MTAG obtained IBD significant SNPs. Table S2: MTAG obtained CD significant SNPs. Table S3: MTAG obtained UC significant SNPs. Table S4 CPASSOC obtained IBD–AS significant SNPs. Table S5: CPASSOC obtained CD–AS significant SNPs. Table S6: CPASSOC obtained UC–AS significant SNPs. Table S7: Shared genes. Table S8: MR analysis results.
